# Supplementary figures and images for: Estimating the impact of climate change on the potential distribution of Indo-Pacific humpback dolphins with species distribution model
Source: PeerJ. 2021 Aug 17;9:e12001. doi: 10.7717/peerj.12001 (PMC8378342; doi:10.7717/peerj.12001)

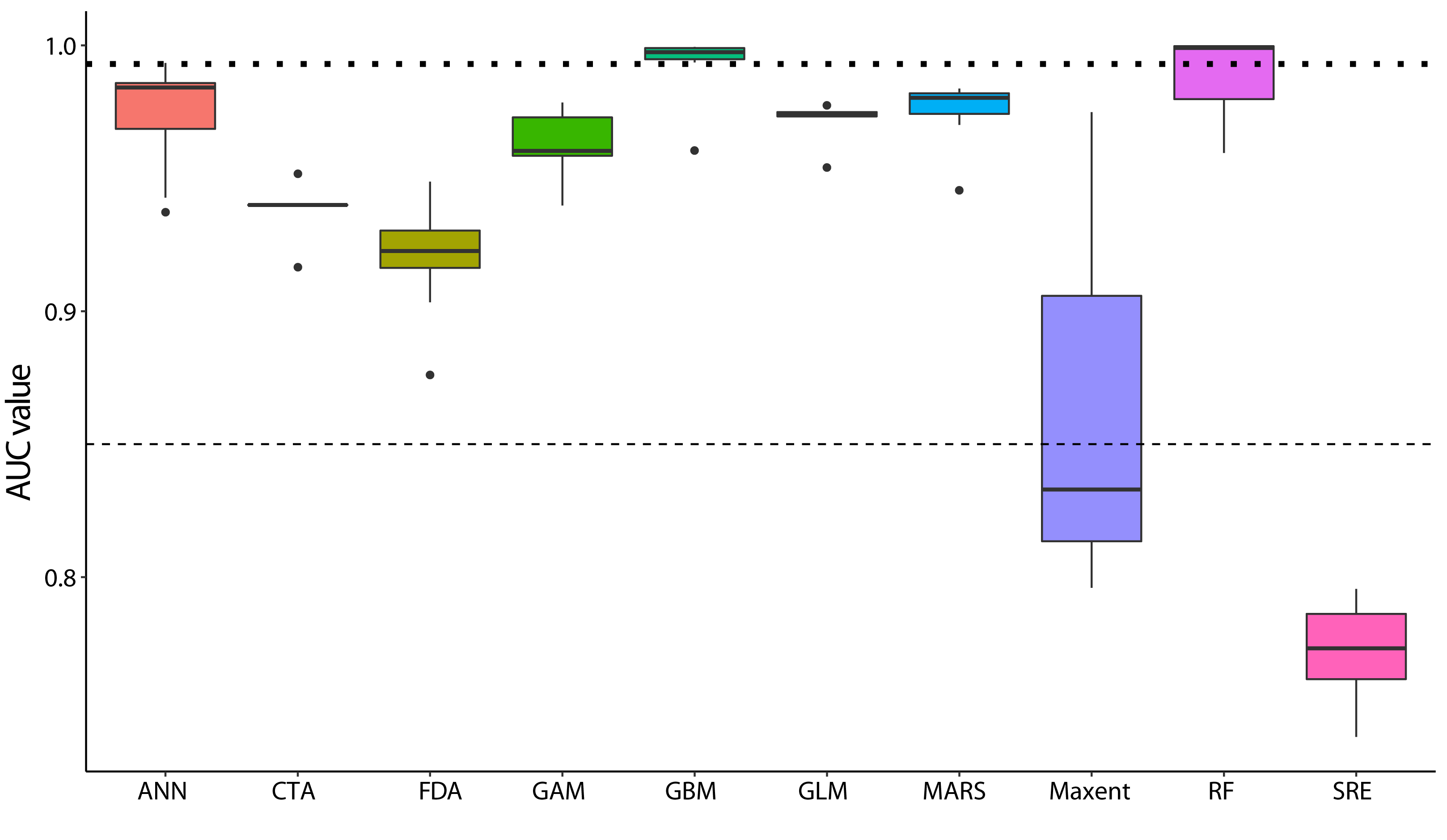

Supplement: Supplemental Information 5 — Dashed line represents the threshold for AUC (0.85) to build the ensemble model. Dotted line represents the AUC value of the ensemble model. [file peerj-09-12001-s005.png]

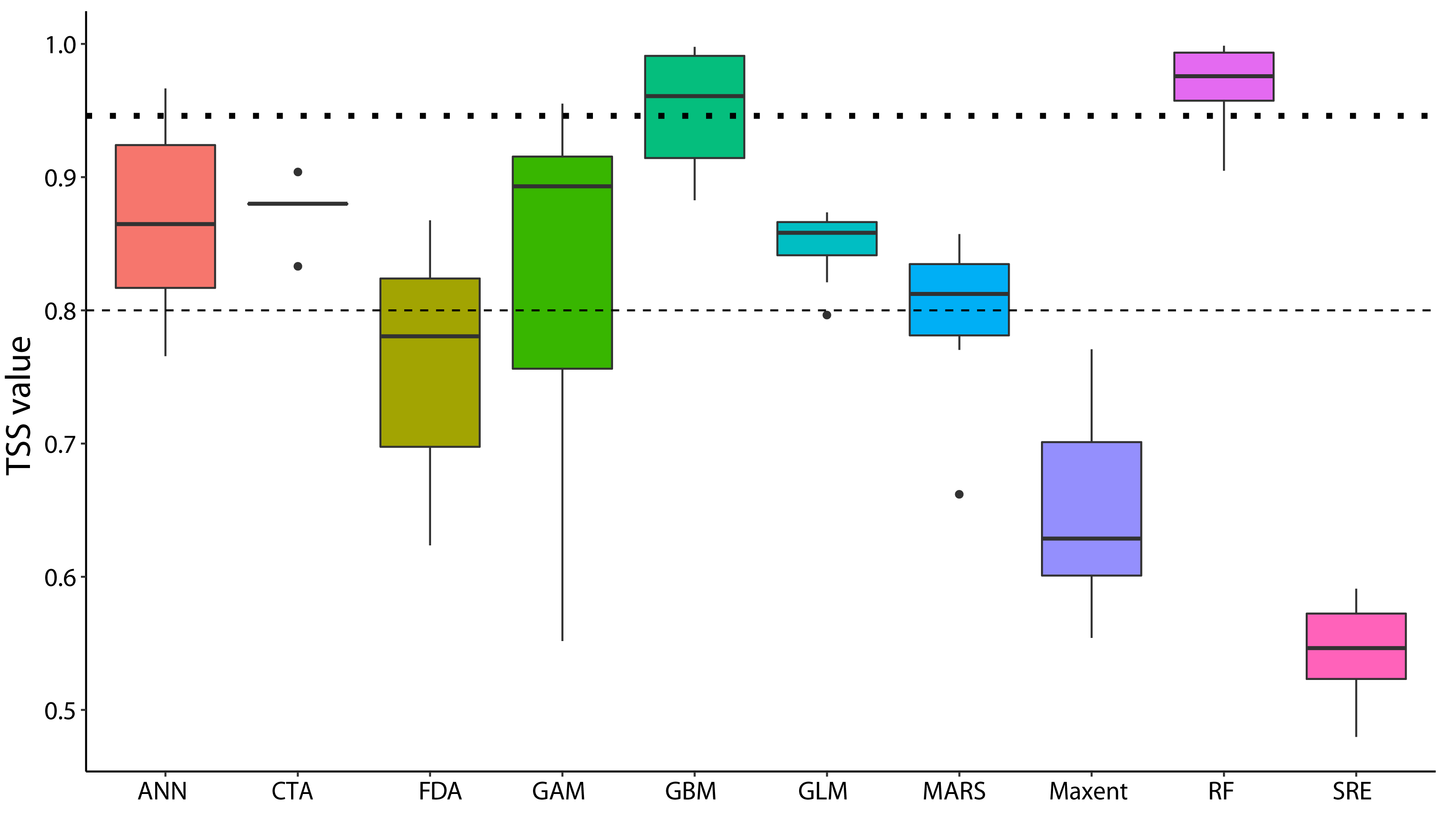

Supplement: Supplemental Information 6 — Dashed line represents the threshold for TSS (0.8) to build the ensemble model. Dotted line represents the AUC value of the ensemble model. [file peerj-09-12001-s006.png]

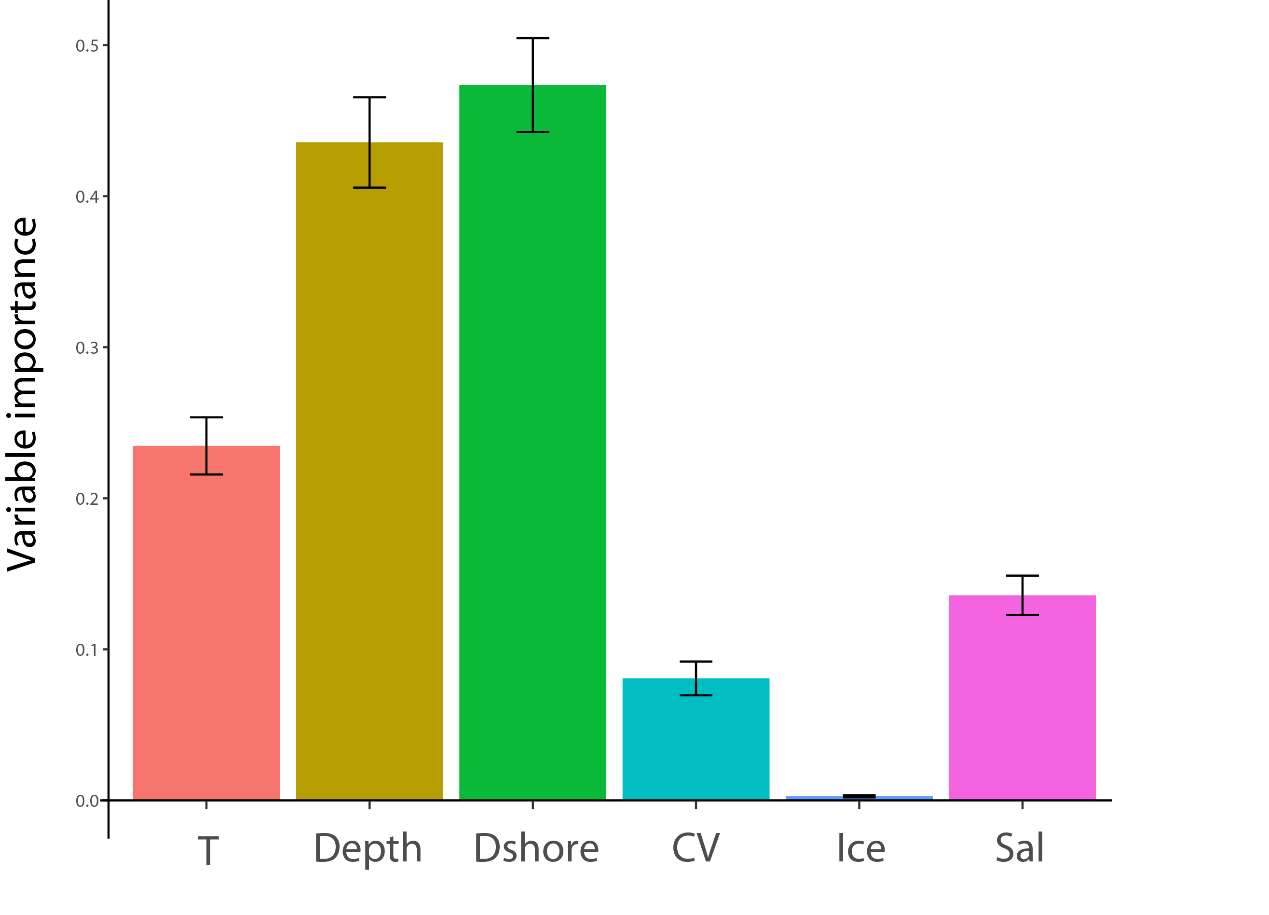

Supplement: Supplemental Information 7 — T: temperature, Depth: ocean depth, Dshore: distance to shore, CV: current velocity, Ice: ice thickness and Sal: salinity. Data are expressed as means ± standard error. [file peerj-09-12001-s007.png]
